# Oral Applications of Cyanoacrylate Adhesives: A Literature Review

**DOI:** 10.1155/2019/8217602

**Published:** 2019-03-17

**Authors:** Eduardo Borie, Eduardo Rosas, Gisaku Kuramochi, Sophia Etcheberry, Sergio Olate, Benjamin Weber

**Affiliations:** ^1^Research Centre in Dental Sciences (CICO), Dental School, Universidad de La Frontera, Temuco, Chile; ^2^Dental School, Faculty of Dentistry, Universidad Finis Terrae, Santiago, Chile; ^3^Universidad del Desarrollo, Concepción, Chile; ^4^Division of Oral and Maxillofacial Surgery, Universidad de La Frontera, Temuco, Chile

## Abstract

Cyanoacrylate adhesives have been used in medicine and dentistry with some controversial opinions. The aim of this review was to summarize the relevant literature regarding the use of cyanoacrylate adhesives for oral wounds during dental and surgical procedures, with focus on the applications, indications, advantages, and disadvantages. In conclusion,* in vivo* and clinical studies have demonstrated in the last few years convincing results regarding the safety, efficacy, ease of application, and feasibility of all types of cyanoacrylate adhesives used in intra- and extraoral procedures.

## 1. Introduction

In the last few years, tissue adhesives have been studied as a good alternative to conventional sutures, although the following properties, among others, are imperative: adequate adhesive strength, appropriate polymerization in a moist environment, biocompatibility, stability, and good working time [1, 2]. In this regard, cyanoacrylate adhesives seem to be a good option for use in medicine as well as dentistry [3]. In August 1998, the U.S. Food and Drug Administration approved the use of a cyanoacrylate adhesive for surgical and trauma wounds [4]. These adhesives polymerize in the presence of hydroxyl ions present in moist tissue, achieving slightly exothermic polymerization [5].

Cyanoacrylates are available in several different forms based on the length and complexity of their chains; these include methyl, ethyl, n-butyl, isoamyl, isohexyl, and octyl cyanoacrylates [6].

Herod [7] developed the first review of the use of cyanoacrylate adhesives in dentistry; however, an update is necessary with* in vitro*,* in vivo*, and clinical researches that include new tests in combination with the use of commercial cyanoacrylate adhesives, which have achieved continuous improvements in the last few years. Then, in the present review, we attempt to summarize the relevant literature regarding the use of cyanoacrylate adhesives for oral wounds created during dental and surgical procedures, with focus on the applications, indications, advantages, and disadvantages.

### 1.1. Applications

The literature reports the widespread use of cyanoacrylate adhesives during procedures performed in various fields of medicine, including gynecology, gastroenterology, neurosurgery, orthopedics, plastic surgery, dermatology, urology, and vascular and cardiac surgery [8]. Common indications for use include esophageal fistula closure, myocardial surgery, bilateral mammoplasty, skin wound closure, bone and cartilage grafting, corneal surgeries, varicose vein occlusion, and embolization of arteriovenous malformations [9–11]. However, the usefulness of this material in the field of dentistry remains unclear, because there are several procedures that allow its application.

Mehta et al. [12] conducted one of the first studies on the use of a cyanoacrylate adhesive in dental surgery. The authors used butyl cyanoacrylate during osteosynthesis of ten mandibular fractures with follow-up periods of 1–6 months, observing no adverse effects or chromosomal changes in the patients. Salata et al. [13] in a short-term period (4–8 days) analyzed the gene expression and the variations of mineralized tissue of autogenous grafts fixed with n-butyl-cyanoacrylate in the mandible of six rabbits and compared this with screw fixation. It was identified that the cyanoacrylate adhesive caused less inflammation and induced a higher mineralized tissue volume than the screw. However, a study in 24 rabbits with 40 days as an evaluation period, in which an adhesive of n-butyl-cyanoacrylate was applied to bond a block of autogenous bone graft to the lateral aspect of the mandible and then analyzed histomorphometrically, demonstrated that the bone graft was maintained fixed during the whole observation period (3-7-20-40 days) but this was not biologically incorporated into the recipient site [14]

Other authors [15, 16] used this material in syringes as well as impregnated in gauze to perform alveoloplasty procedures, while Cooper and Paige [17] demonstrated its utility for cleft lip and cleft palate surgery in adults and children. In addition, Pérez et al. [10] reported the use of a cyanoacrylate adhesive in more than 100 patients who underwent exodontia or apical/periodontal surgeries, with no adverse effect in any case. Also, Nevins et al. [18] used a cyanoacrylate adhesive as a protective layer of collagen exposed membrane for extraction socket preservation.

In the field of periodontics, Rezende et al. [9] reported a case using the cyanoacrylate adhesive for fixing a resorbable membrane to the recipient bone in a vertical defect to allow guided tissue regeneration, showing esthetical results four years after the procedure, while Pérez [10], in a prospective study, used this adhesive in 19 patients for fixing mucogingival grafts, identifying a good tolerance. In addition, the cyanoacrylate adhesive has been used in free gingival grafting [19–21], apicectomy [10], root sectioning [22], and bonding of fractured tooth fragments [23]. Kulkarni et al. [24] reported the use of cyanoacrylate in periodontal pockets during surgical procedures involving periodontal flaps in 24 individuals, Ozcan et al. [25] used it to promote palatal wound healing after free gingival graft harvesting, and Ranson et al. [26] used it for stabilizing pedicle grafts during soft tissue surgeries.

In the field of oral and maxillofacial surgery, Choi et al. [27] evaluated the usefulness of a cyanoacrylate adhesive for the closure of maxillary sinus membrane perforations in rabbits by using histological analysis. The authors identified complete healing of the Schneiderian membrane without any signs of inflammation. Bozkurt and Saydam [28] used cyanoacrylate adhesives for wound closure after head and neck surgeries of 80 patients, reporting no complications and a high satisfaction of the patients related to scarring when this adhesive was used, while Sagar et al. [5] used them for intraoral wounds closure such as mucosal incision, biopsies, fractures, adenoma excision, and apical surgery. These adhesives have also been used as hemostatic agents in high-risk extraction cases, such as those involving third-molar removal [29–31].

In the field of esthetic surgery, cyanoacrylate adhesives have been used for wound closure and in skin grafting procedures, blepharoplasty, face and brow lifts, and other cosmetic surgeries [32]. Moreover, they have been used for the management of lacerations in children in order to avoid anesthetic procedures [33]. In general, cyanoacrylate can be used in any region of the body where there is no tension [32].

### 1.2. Contraindications

Contraindications for the use of cyanoacrylate adhesives are limited. Specifically, they cannot be used in areas of tension, such as joints, areas subjected to friction, and areas showing infection and/or contamination with exudate [34–37]. In addition, they cannot be used in conjunctival procedures and patients with allergy to cyanoacrylate [5, 8, 32, 38].

### 1.3. Advantages

The literature reports several advantages of cyanoacrylate adhesives with regard to their use in oral surgery procedures.

**Figure 1:**
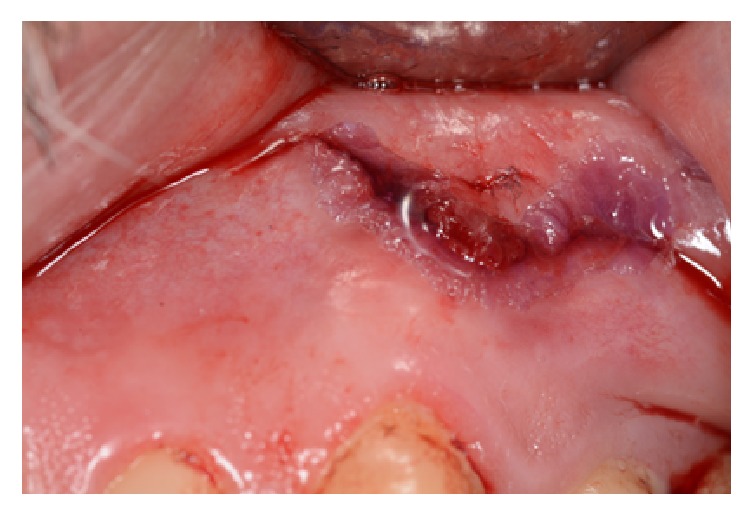
Wound closure in a moist environment during an apical surgery with an adhesive composed of n-butyl-cyanoacrylate and octyl cyanoacrylate.

Cyanoacrylate adhesives become hard in the presence of fluids such as blood or saliva (Figure 1), with good biodegradability and hemostatic and bacteriostatic properties [1, 3, 9, 22, 32]. The simple and fast application of cyanoacrylate adhesives, along with their good hemostatic properties, allows the satisfactory closure of oral mucosal wounds [9, 22, 32, 39]. Al-Belasy and Amer [40] confirmed the hemostatic effects of cyanoacrylate in patients who underwent oral surgeries while receiving warfarin treatment, observing satisfactory healing without complications.

In addition, cyanoacrylate adhesives provide a mechanical barrier that prevents detritus collection, thus reducing the healing time and accelerating epithelial keratinization [32, 39]. Patients have also reported high satisfaction levels for this material [5, 20, 32].

In cases of guided bone regeneration, cyanoacrylate adhesives simplify membrane fixation and reduce time and discomfort for the clinician [9]. Gümüs and Buduneli [19] reported less shrinkage of free gingival grafts when they used a cyanoacrylate adhesive, identifying lesser dimensional changes, clinical time, and pain at the recipient site with cyanoacrylate than with microsurgical (suture: 7.0, magnifying) and conventional (suture: 5.0) techniques.

Because cyanoacrylate adhesives do not require any needles for application, they eliminate the risk of puncture accidents for both clinicians and auxiliary personnel [32, 40, 41].

The pain-free application procedure for these adhesives allows their use in cases where the anesthetic effect has diminished [5, 10, 36, 37] and for anxious or fearful patients [42] such as children. Moreover, cyanoacrylates do not exhibit genotoxic activity or systemic toxicity, and they do not cause mucosal irritation or cutaneous sensitivity [4, 8, 10, 43]. Researchers have also reported minimal inflammatory response in regions of use, with zero potential for necrosis [2, 17, 44, 45]. Finally, some authors have reported excellent cosmetic results without dehiscence or allergic reactions [17, 44, 46, 47].

### 1.4. Disadvantages

Not many disadvantages of cyanoacrylate adhesives have been discussed in the literature, although the high cost of octyl and isoamyl compounds [8] and reduced tensile strength [5] have been reported.

### 1.5. Carcinogenicity and Toxicity

Till date, there is no sufficient evidence showing that cyanoacrylates are carcinogenic for humans [2, 35].

The toxicity of cyanoacrylates remains a controversial topic. Avery and Ord [34] described good tolerance of tissues to butyl cyanoacrylate and reported that all cyanoacrylates except methyl cyanoacrylate allow for satisfactory healing without necrosis and promote the proliferation of connective tissue. Some authors have reported concerns about ethyl cyanoacrylate [8, 48, 49] causing skin toxicity, necrosis, and allergic dermatitis. These authors stated that an increase in the number of lateral chains in the cyanoacrylate molecule decreases the degree of cytotoxicity, increases the healing time, and reduces its adhesiveness. However, some researchers have reported that ethyl cyanoacrylate is a safe and inexpensive adhesive that aids in joining of wound edges, results in an acceptable inflammatory response with decreased polymorphonuclear infiltration and an esthetically acceptable scar, and does not cause necrosis or allergic reactions [8, 11, 20, 45].

Several studies have evaluated the toxicity of cyanoacrylate adhesives in rats. One author applied ethyl cyanoacrylate adhesive to the skin of rats and saw no signs of toxicity [43], while other authors applied butyl cyanoacrylate over cuts in the oral mucosa of rats and did not observe any kidney or liver toxicity [6]. However, some authors [35, 37, 50] have also reported that the toxicity is related to the heat released during the polymerization reaction and the presence of unreacted monomers; moreover, there is a direct relationship between the degree of toxicity and the length of the alkyl chain. These reports require further studies for clarification.

### 1.6. Bacteriostatic and Hemostatic Properties

Cyanoacrylate adhesives exhibit good bacteriostatic properties [34, 35] that are explained by the strong electronegative charge of the polymer and its ability to form a mechanical barrier that prevents the entry of any material or organisms [37]. In addition, butyl cyanoacrylate adhesives have been reported to exhibit antibacterial effects on Gram-positive organisms [1, 2].

**Figure 2:**
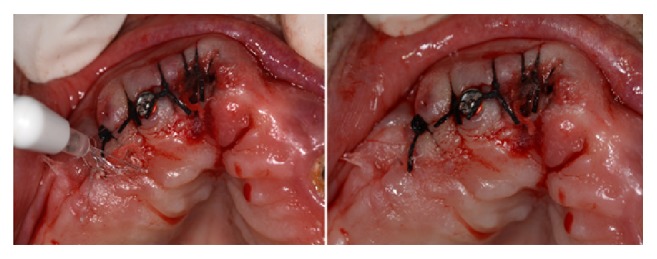
Application of ethyl cyanoacrylate adhesive during a dental implant placement procedure to achieve immediate hemostasis in a patient that uses anticoagulant therapy.

Cyanoacrylate adhesives also provide immediate hemostasis on application [2, 37, 43]; this is attributed to their ability to form a mechanical barrier at the wound site, which favors the coagulation process and allows hemorrhage control [39] (Figure 2).

### 1.7. Cyanoacrylate Adhesives versus Sutures for Intraoral Wounds

Currently, suturing is the most commonly used method for intraoral wound closure; however, reported disadvantages have fueled the search for new methods and materials. In general, wounds show great potential for reinfection during the healing process, which is frequently observed in the oral cavity because of biofilm formation and food accumulation that are further aggravated by the presence of sutures [2]. Suturing requires anesthesia and needles, a significantly longer duration, and a second visit for suture removal [1, 2, 36], thus increasing patient discomfort [17, 33] and the risk of puncture accidents for clinicians and auxiliary personnel [2, 41].

Some* in vivo* studies have compared sutures with different types of cyanoacrylate adhesives in animals and humans, and they concluded that cyanoacrylates are superior to sutures. Kulkarni et al. [24] compared a butyl cyanoacrylate adhesive with sutures for intraoral wound closure in rabbits though clinical and histological evaluations at 7 and 21 days after the procedure. They identified an agreement between clinical and histological parameters and noted that hemostasis was faster and the procedural duration was shorter with butyl cyanoacrylate than with sutures. Moreover, at 7 days, there was more inflammatory infiltration at the sutured sites, although complete epithelization without alterations, irregular connective tissue, and the same amount of vascularization was observed with both methods. Even at 21 days, organization of the connective tissue, epithelization, vascularity, and inflammatory infiltration were comparable between methods.

Cooper and Paige [17] evaluated the usefulness and safety of octyl cyanoacrylate adhesives used for skin closure in 18 adult and pediatric patients with cleft lips, and they reported excellent cosmetic results with no allergic reactions, infections, or dehiscence. In addition, they mentioned that the elimination of a second visit for suture removal was an added advantage, particularly for children.

Joshi et al. [36] compared sutures with an isoamyl cyanoacrylate adhesive for wound closure after third-molar disimpaction procedures in 30 patients. Pain during the first 3 days after surgery and bleeding were significantly lesser with the cyanoacrylate adhesive than with sutures. The authors concluded that cyanoacrylate adhesives are associated with minimal inflammation and tissue manipulation when compared with sutures.

Vastani and Maria [15] conducted a study including 30 patients who underwent an alveoloplasty procedure with suturing on one side and isoamyl cyanoacrylate application on the other side. Clinical and histological examinations were performed at 1, 7, 14, and 21 days after surgery. For up to 14 days, sensitivity and erythema were significantly lesser with the cyanoacrylate than with sutures, whereas there were no differences at 21 days. No patient showed infection or wound dehiscence. Histological analysis of biopsy specimens obtained at 7 and 14 days after surgery showed significantly greater inflammatory cell infiltration and vascularity on the sutured side. These findings indicated that inflammation after wound closure is greater with sutures than with cyanoacrylate adhesives.

Soni et al. [4] performed a randomized clinical trial comparing sutures with an octyl cyanoacrylate adhesive for wound closure in the maxillofacial region of 29 patients. The evaluated variables included the time required for wound closure, postoperative complications, and esthetic outcomes. The average time required for wound closure was significantly lesser in the cyanoacrylate group (69.5±33.39 s) than in the suture group (379.00±75.39 s), with no differences in the rate of postoperative complications and esthetic outcomes between the two groups.

Another controlled clinical trial [10] of patients who underwent oral surgeries compared the efficacy of a butyl cyanoacrylate adhesive used for 130 patients with that of sutures used for 30 patients. The wounds were monitored for hemostasis, dehiscence, inflammation, and infection during the first 30 min and at 7–9 days after surgery. Hemostasis was immediately achieved in the cyanoacrylate group and at 20–30 min in the suture group. There were no differences in the healing and complication rates between the two groups. The authors concluded that butyl cyanoacrylate achieves rapid hemostasis, is easy to apply, and avoids the need for a second visit because the adhesive peels off after 7 days and does not cause intraoral tissue reactions other than the normal healing response.

A randomized clinical trial [51] performed in 36 patients, in which the n-butyl/n-octyl cyanoacrylate adhesives were compared with 6-0 polytetrafluoroethylene (PTFE) sutures in terms of patient-centered outcomes, showed only statistical differences in the time required, while no differences were identified regarding the discomfort, analgesic intake, pain level, and the modified early-wound healing index.

Gümüs and Buduneli [19] compared three different stabilization methods, namely, cyanoacrylate adhesive fixation, conventional suturing, and microsurgery, with regard to the amount of free gingival graft shrinkage at baseline and at 1, 3, and 6 months after surgery in 45 patients. At all time points, the cyanoacrylate group showed significantly lesser graft contraction than did the other groups, with the least working time, no bleeding complications, and lesser pain in the first week after surgery.

Giray et al. [22] performed clinical and microscopic assessments of mucosal wounds closed with sutures or a butyl cyanoacrylate adhesive in 15 patients who underwent root resection in the anterior teeth. They placed an incision on both sides of the labial frenum and closed one with sutures and the other with the cyanoacrylate adhesive. Clinical examinations were performed at 1, 2, 3, 7, 14, and 21 days after surgery, while biopsy specimens from both sides were obtained and examined under a transmission electronic microscope at 7 days. On days 1 and 2, pain and edema were significantly greater on the sutured side, while, on days 3, 7, and 14, the two sides showed no significant differences. However, on day 21, scar formation was markedly greater on the sutured side. Microscopic examinations revealed a structurally normal morphology in both specimens, with no morphological differences in the epithelial layers except the stratum spinosum, which showed marked invaginations in the specimen from the cyanoacrylate group. In addition, cellular structures in both specimens were normal in all aspects.

## 2. Conclusion

In summary,* in vivo* and clinical studies have demonstrated convincing results regarding the safety, efficacy, ease of application, and feasibility of all types of cyanoacrylate adhesives used in intra- and extraoral procedures.
